# Analysis of coevolution in nonstructural proteins of chikungunya virus

**DOI:** 10.1186/s12985-016-0543-1

**Published:** 2016-06-02

**Authors:** Jaspreet Jain, Kalika Mathur, Jatin Shrinet, Raj K. Bhatnagar, Sujatha Sunil

**Affiliations:** Insect Resistance Group, International Centre for Genetic Engineering and Biotechnology, New Delhi, 110067 India

**Keywords:** Chikungunya virus, Coevolution, Nonstructural proteins, Eukaryotic linear motifs

## Abstract

**Background:**

RNA viruses are characterized by high rate of mutations mainly due to the lack of proofreading repair activities associated with its RNA-dependent RNA-polymerase (RdRp). In case of arboviruses, this phenomenon has lead to the existence of mixed population of genomic variants within the host called quasi-species. The stability of strains within the quasi-species lies on mutations that are positively selected which in turn depend on whether these mutations are beneficial in either or both hosts. Coevolution of amino acids (aa) is one phenomenon that leads to establishment of favorable traits in viruses and leading to their fitness.

**Results:**

Fourteen CHIKV clinical samples collected over three years were subjected to RT-PCR, the four non-structural genes amplified and subjected to various genetic analyses. Coevolution analysis showed 30 aa pairs coevolving in nsP1, 23 aa pairs coevolving in nsP2, 239 in nsP3 and 46 aa coevolving pairs in nsP4 when each non-structural protein was considered independently. Further analysis showed that 705 amino acids pairs of the non-structural polyproteins coevolved together with a correlation coefficient of ≥0.5. Functional relevance of these coevolving amino acids in all the nonstructural proteins of CHIKV were predicted using Eukaryotic Linear Motifs (ELMs) of human.

**Conclusions:**

The present study was undertaken to study co-evolving amino acids in the non-structural proteins of chikungunya virus (CHIKV), an important arbovirus. It was observed that several amino acids residues were coevolving and shared common functions.

## Background

Coevolution at the molecular level is an established phenomenon exhibited by organisms in order to optimize their physiological performance and serve as an effective determinant of fitness [1, 2]. Coevolution refers to synchronized changes that organisms or proteins undergo and can occur with respect to pathogen-host [3], as well as within the organism [4, 5], often revealing functional coordination regarding the interacting pairs. One of the most compelling examples of coevolution of host and pathogen can be witnessed in the retrovirus, mainly because of their tolerance to mutagenesis [6]. Studies in HIV have shown that drug resistance is manifested by coevolution of proteins in the virus under drug selective pressure [7, 8]. Selection advantage of coevolution may ultimately result in recombination events [9].

Chikungunya virus (CHIKV) is a re-emerging arbovirus belonging to family Togaviridae. Known to exist as mixed populations of genomic variants, known as quasispecies, these variants confer phenotypic plasticity and adaptability to new environments [10, 11]. In spite of this, strong purifying selection plays a role in evolution of arboviruses [12]. Owing to its complicated life cycle that includes its survival in two disparate hosts, one invertebrate and the other vertebrate, several studies have be devoted to understand arbovirus fitness determinants [13–15] including host-specific fitness benefits [16, 17].

Owing to their small genome sizes, viruses lack the machinery for their replication and other processes and are mostly dependent on the host for these functions. In order to perform their functions, viruses have mastered motif mimicry based hijacking strategy wherein small motifs of the virus mimic host protein motifs. Recognized as Eukaryotic Linear Motif (ELM) or mini motif or Short Linear Motif (SLiM), these linear motifs function independent of the tertiary structure of the host protein and are also needed for post-translational modifications, cell signaling, cellular trafficking and play important role in virus maturation [18, 19].

A recent study describes homologous recombination in CHIKV where non-structural protein nsP3 is reported to be site for recombination [20]. The current study was undertaken to study the coevolving aa in the replication machinery of CHIKV, namely the non-structural proteins, nsP1, nsP2, nsP3 and nsP4. Further, using Eukaryotic Linear Motifs (ELM) [18], we predicted the functional relevance of the coevolving aa pairs. The results obtained in the present study revealed important intra-molecular coevolving aa residues of the replication complex of CHIKV. The study provided insights to functional significance of some of the coevolving aa with respect to human protein motifs.

## Methods

### Sample collection

Fourteen patients from Safdarjung Hospital, New Delhi were recruited for the present study after obtaining their informed consent (Table 1). Blood was drawn from these patients, sera separated and subjected to ELISA for testing the presence of chikungunya virus IgM antibodies using IgM capture ELISA kits supplied by National Institute of Virology (NIV), Pune. The sera samples were amplified once in C6/36 cells and once in VERO cells.

**Table 1:** Details of chikungunya samples used in the study

CHIKV ID	Year of sample collection	IgM status	RT-PCR
IND-10-DEL1	2010	Negative	Positive
IND-10-DEL2	2010	Negative	Positive
IND-10-DEL3	2010	Negative	Positive
IND-10-DEL4	2010	Negative	Positive
IND-10-DEL5	2010	Negative	Positive
IND-10-DEL6	2010	Negative	Positive
IND-10-DEL8	2010	Positive	Positive
IND-10-DEL9	2010	Negative	Positive
IND-10-DEL10	2010	Negative	Positive
IND-10-DEL11	2010	Positive	Positive
IND-10-DEL12	2010	Negative	Positive
IND-11-DEL01	2011	Negative	Positive
IND-12-DEL02	2012	Negative	Positive
IND-12-DEL15	2012	Not Done	Positive

### VNA extraction, RT-PCR, cloning and plasmid sequencing

Viral nucleic acid (VNA) was extracted from the stored samples using High Pure Viral Nucleic Acid kit (Roche, Germany). Non-structural genes of CHIKV (nsP1, nsP2, nsP3 and nsP4) were amplified individually using Titanium One–step RT-PCR Kit (Clontech, USA), using primers listed in Table 2. The whole genome of CHIKV was amplified for three samples using gene specific primers (Table 2). The amplified products were purified using SureExtract PCR clean up/gel extraction kit (Nucleopore, India), cloned in pGEM-T Easy vector and sequenced. The sequences have been submitted to NCBI (GenBank) with Accessions numbers KU365282-KU365292.

**Table 2:** Primers used for gene amplification

Primer name	Sequence
nsP1 FP	GTAATGGATCCTGTGTACGTGG
nsP1 RP	TGCACCCGCTCTGTCCT
nsP2 FP	GTAATGGGAATAATAGAGACTCCGAGA
nsP2 RP	TCCTGCTCGGGTGGCCTG
nsP3 FP	GTAATGGGATGTGCACCGTCGTACCGG
nsP3 RP	TTACTCGTCGTCCGTGTCTG
nsP4 FP	GTAATGGGACGACTAGACAGGGCAGGTG
nsP4 RP	AGGACCGCCGTACAAAGTTA
E1 FP	GTAATGGCGTACGAACACGTAACAG
E1 RP	TTAGTGCCTGCTGAACGACAC
E2 FP	GTAATGGGAAGCACCAAGGACAACTTCAAT
E2 RP	TTTAGCTGTTCTGATGCAGC
E3 FP	GTAATGGGATGGAGTCTTGCCATCCCAGT
E3 RP	GCGTCGCTGGCGGTGG
Capsid FP	GTAATGGAGTTCATCCCAACCC
Capsid RP	CTCTTCGGCTCCCTCAG
6K FP	GTAATGGCGGCCACATACCAAGAG
6K RP	GCTCACAGTGTGGGCAC

### Sequence and phylogenetic analysis

CHIKV gene sequences were trimmed and taken for further analysis. Phylogenetic analysis of 14 sequences of Delhi samples and 195 available complete genome and non-structural polyprotein sequences (nsP1, nsP2, nsP3 and nsP4) of CHIKV was performed using MEGA version 6 [21] MAFFT [22] was used to perform multiple aa sequence alignment. For the construction of phylogenetic tree, neighbor-joining algorithm and Poisson distribution distance model were utilized. Reliability of the analysis was evaluated using bootstrap test with 1500 replications. Amino acid sequence analysis was performed on all the nonstructural proteins of all Delhi strains and compared with the sequences from India and rest of the world available in various public databases.

### Molecular sequence evolution analysis

All analysis involved 196 aa sequences (consisting of one consensus sequence representing all Delhi samples and 195 already available sequences). MEGA 6 software was used to conduct Evolutionary analyses using the Poisson correction model. The rate variation among sites was modeled with a gamma distribution (shape parameter = 1). Substitution pattern and rates were estimated under the Jones-Taylor-Thornton [23] model (+G). A discrete Gamma distribution was used to model evolutionary rate differences among sites (5 categories, [+G]). Mean evolutionary rates of substitutions per site in these categories and the aa frequencies were also calculated. For estimating Maximum likelihood (ML) values, a tree topology was automatically computed. The maximum Log likelihood for this computation was also calculated. Nucleotide diversity and Tajima test statistic [24] were deduced using MEGA6 and the selection pressure on polyprotein was deduced.

### Coevolution analysis

Coevolution analysis of the replication complex of chikungunya virus was done using Coevolution analysis using Protein Sequences (CAPS) [25] software (considering default setting for intra-molecular coevolution analysis without making use of any structural information (as there is no structural information present for chikungunya virus proteins in the public domain). It was used to identify groups of coevolving pairs with correlation coefficient >0.5 (Correlation Value). All the aa within each group coevolving with all the others within the same group were identified. Cytoscape [26] was used to generate networks of coevolving aa sites.

### Eukaryotic Linear Motifs (ELM) analysis

Eukaryotic Linear Motifs (ELM) of all the non-structural proteins of chikungunya virus (CHIKV) were predicted separately using Eukaryotic Linear Motif resource server (ELM). As the virus purified from clinical samples were sequenced and used in this study, we predicted human’s linear motifs in CHIKV non-structural proteins. The ELM GO terms were retrieved from the ELM database and were used for predicting functional significance of the coevolving pairs. Currently, the database has 1594 ELM instances of *Homo sapiens* and GO terms related to these instances. The motif probability cutoff was taken as 100 (default value) and *Homo sapiens* was selected as the preferred species to predict the conserved peptide linear motif of human in chikungunya virus non-structural proteins. The motifs were filtered based on cell compartment terms. The ELMs with p-value ≤ 0.001 were considered for further analysis.

## Results

Patient samples were collected from Delhi region, India for three consecutive years 2010-2012. The year 2010 saw a major outbreak of chikungunya in Delhi following which there has been a steady decline of cases. The 14 samples used in this study were mainly IgM negative (*n* = 11), while two samples were IgM positive and IgM status was not available for one sample. All the 14 samples were positive for CHIKV by Real-Time Quantitative Reverse Transcription PCR (qRT-PCR). The samples were passaged once each in C6/36 and VERO cells; the nsPs amplified end to end, cloned in pGEM-T easy vectors and sequenced using Sanger’s dideoxy sequencing. The sequences were trimmed and aligned with 195 nsPpolyprotein sequences from the Genbank database. A total of 29 sequences from Indian strains and 166 sequences from across the globe were taken for further analysis.

### Sequence and phylogenetic analysis of Delhi samples

Amino acid sequence analysis was performed on the complete gene sequences of nsP1 (535aa), nsP2 (798aa), nsP3 (530aa), nsP4 (611aa). It was observed that all the sequences were high level of identity (99 %) amongst each other. With respect to the East/Central/South African (ECSA) prototype, S27, several variations were observed in the Delhi sequences. The nsPs sequences from other parts of the country, namely, Andhra Pradesh, Gujarat, Karnataka, Kerala, Maharashtra, Rajasthan, Tamil Nadu and West Bengal were also taken for this analysis. In-depth aa sequences analysis highlighted a total of 25 variations in the non-structural proteins. Details of all the mutations are provided in Table 3a-d.

**Table 3:** Amino acid variations in Delhi samples in comparison with ESCA lineage and other Indian samples

a: Amino acid variations of Delhi samples in nsP1												
Name of protein	Sample Site							128	253	314	376	488
CHIKV nsp1	S27- African Prototype							T	K	M	T	Q
	Andhra Pradesh							K	K	L/M	M	R
	(*n* = 5)											
	Gujarat							K	K	L/M	M	R
	(*n* = 5)											
	Karnataka							K	K	L	M	R
	(*n* = 3)											
	Kerala							K	K	L/M	M	R
	(*n* = 9)											
	Rajasthan							K	K	M	M	R
	(*n* = 2)											
	Tamil Nadu							K	K	M	M	R
	(*n* = 1)											
	West Bengal							T	T	M	T	Q
	(*n* = 1)											
	Maharashtra							T/K	T/K	M	M	R
	(*n* = 3)											
	Delhi							K	K	M	M	R
	(*n* = 14)											
b: Amino acid variations of Delhi samples in nsP2												
Name of protein	Sample Site	48	54	181	237	238	329	374	411	539	708	793
CHIKV nsp2	S27- African Prototype	V	N	V	L	L	K	H	N	L	L	A
	Andhra Pradesh	V	N	V	L	L	K	Y	N	L	L	V
	(*n* = 5)											
	Gujarat	V	N	V	L	L	K	Y	N	L	L	V
	(*n* = 5)											
	Karnataka	V	N	V/A	L	L	K	Y	N	L	L	V
	(*n* = 3)											
	Kerala	A/V	N	V	R/L	P/L	E/K	Y	D/N	L/S	P/L	V
	(*n* = 9)											
	Rajasthan	V	N	V	L	L	K	Y	N	L	L	V
	(*n* = 2)											
	Tamil Nadu	V	N	V	L	L	K	Y	N	L	L	V
	(*n* = 1)											
	West Bengal	V	S	V	L	L	K	H	N	L	L	A
	(*n* = 1)											
	Maharashtra	V	S/N	V	L	L	K	H/Y	N	L	L	V/A
	(*n* = 3)											
	Delhi	V	N	V	L	L	K	Y	N	L	L	V
	(*n* = 14)											
c: Amino acid variations of Delhi samples in nsP3												
Name of protein		Sample Site						175	217	341	353	501
CHIKV nsp3		S27- African Prototype						V	Y	T	I	L
		Andhra Pradesh						I	H	T/M	I	L
		(*n* = 5)										
		Gujarat						I	H	T	I	S/L
		(*n* = 5)										
		Karnataka						I	H	T	I	S/L
		(*n* = 3)										
		Kerala						I	H	T	I	L
		(*n* = 9)										
		Rajasthan						I	H	T	I	L
		(*n* = 2)										
		Tamil Nadu						I	H	M	I	L
		(*n* = 1)										
		West Bengal						V	Y	T	T	L
		(*n* = 1)										
		Maharashtra						I/V	Y/H	T	I/T	L
		(*n* = 3)										
		Delhi						I	H	T	I	L
		(*n* = 14)										
d: Amino acid variations of Delhi samples in nsP4												
Name of protein		Sample Site							43	85	90	235
CHIKV nsp4		S27- African Prototype							A	R	S	Q
		Andhra Pradesh							A	R	S	Q
		(*n* = 5)										
		Gujarat							A	R	S	Q
		(*n* = 5)										
		Karnataka							A	R	S	Q
		(*n* = 3)										
		Kerala							A	R	S	Q
		(*n* = 9)										
		Rajasthan							A	R	S	Q
		(*n* = 2)										
		Tamil Nadu							A	R	S	Q
		(*n* = 1)										
		West Bengal							L	K	A	R
		(*n* = 1)										
		Maharashtra							L/A	K/R	A/S	R/Q
		(*n* = 3)										
		Delhi							A	R	S	Q
		(*n* = 14)										

**Fig. 1:**
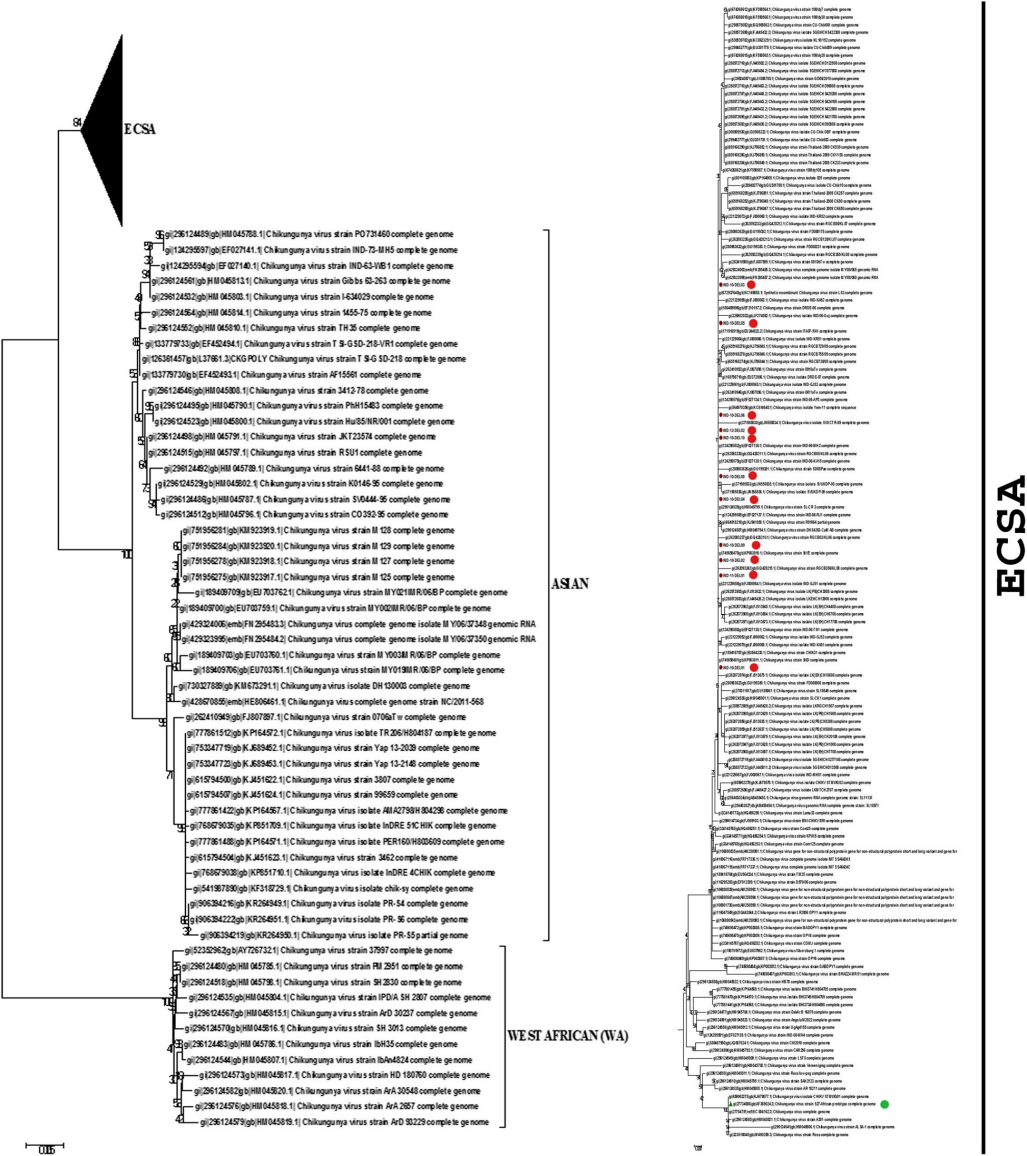
Phylogenetic analysis of nsPs. Total 209 nsP sequences (14 of CHIKV Delhi samples and 195 available in public domain) were aligned using MEGA 6.0 software to construct phylogenetic tree based on Neighbor-joining algorithm and Poisson distribution distance model. Bootstrap values were kept 1500 to ensure reliability. **a** Phylogenetic analysis of the Delhi sequences was done and its placement in CHIKV genotype group was determined. **b** Inset of ECSA clade. All samples used for the study belonged to the ECSA clade. : represent samples from Delhi region and : represents S27- African Prototype used as control for the study

Phylogenetic analysis of the samples for all the nsPs revealed that they belonged to ECSA I subgroup 1 that belongs to Indian Ocean Lineage (IOL) (Fig. 1).

### Estimation of evolutionary parameters based on phylogenetic analysis

Evolutionary parameters of two ECSA subgroups with the Delhi samples were checked using pairwise genetic divergence between groups. The analysis confirmed the occurrence of Delhi samples in the ECSA subgroup 1 as the genetic divergence between the two groups was 0.001 ± 0.000. Similarly the genetic divergence between ECSA subgroup 2 and Delhi samples was observed to be 0.010 ± 0.002.

Furthermore, overall value of the shape parameter for discrete Gamma Distribution was estimated to be 0.2609. Mean evolutionary rates in these categories were 0.00, 0.03, 0.21, 0.83, 3.93 substitutions per site. The aa frequencies are 7.69 % (A), 5.11 % (R), 4.25 % (N), 5.13 % (D), 2.03 % (C), 4.11 % (Q), 6.18 % (E), 7.47 % (G), 2.30 % (H), 5.26 % (I), 9.11 % (L), 5.95 % (K), 2.34 % (M), 4.05 % (F), 5.05 % (P), 6.82 % (S), 5.85 % (T), 1.43 % (W), 3.23 % (Y), and 6.64 % (V). Also, for estimating maximum Log likelihood (ML) values, a tree topology was automatically computed. The ML for this computation was -4169.220. Neutrality of mutation in sequence sequences was determined using Tajima’s Neutrality Test (Table 4). Negative Tajima test statistic signifies an excess of low frequency polymorphisms, indicating population size expansion (e.g., after a bottleneck or a selective sweep) and/or purifying selection.

**Table 4:** Tajima’s neutrality test

M (number of sequences)	S (Number of segregating sites	ps (ps = S/n)	Θ (Θ = ps/a1)	Π (nucleotide diversity)	D (Tajima test statistics)
196	112	0.111888	0.019656	0.011574	-1.29759

### Coevolution analysis

Coevolution analysis was performed using multiple sequence alignment of both individual non-structural proteins (nsPs) and polyprotein of the complete nsPs. A total of 209 sequences, 195 CHIKV sequences present in the public domain and 14 CHIKV sequences obtained from Delhi was used in this analysis. The first round of analysis involved the complete sequences to identify the coevolving pairs. Subsequently, the mutation status of these pairs was examined in the Delhi samples. Details of the analysis for each of the nsPs and the whole polyprotein are described below.

**Fig. 2:**
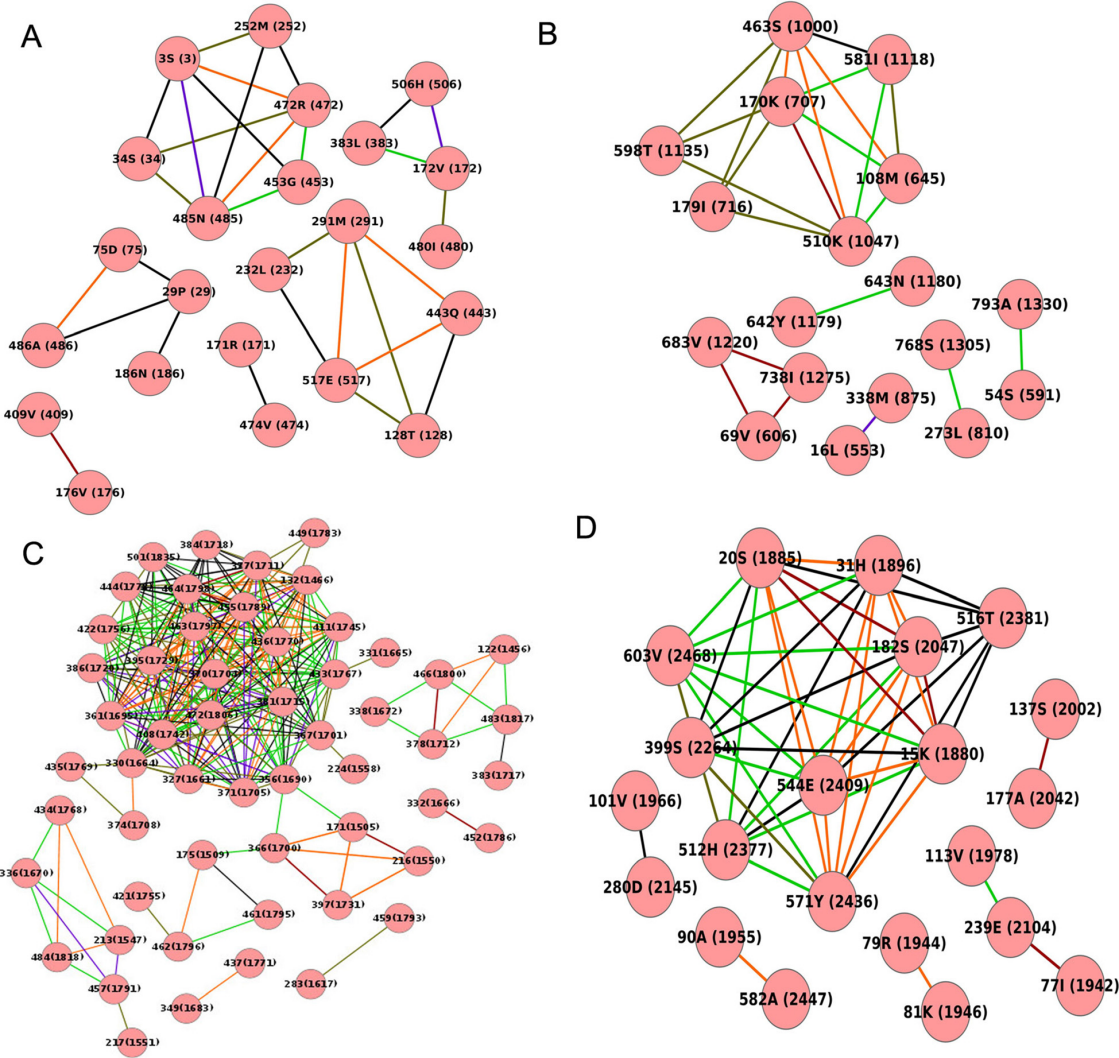
Coevolution analysis of nsPs. **a**-**d** MSA of all CHIKV nsP sequences (total 209) were analyzed using CAPS software to study coevolving aa within individual nsPs. Mutated aa pairs with correlation value above 0.5 were used for construction of network using Cytoscape software. Edge color varies with correlation value: red (1.0), orange (~0.9), purple (~0.8), light green (~0.7), black (~0.6) and dark green (~0.5). Node names include aa residue positions in individual nsP (as well as in polyprotein)
- nsP2: nsP2 has 798 aa (aa) and the whole protein was taken for coevolution analysis. It was observed that 23 aa pairs involving 18 aa residues coevolved at a correlation >0.5, of which, seven aa pairs showed a correlation of 0.9-1.0 (Fig. 2b). Mutations were observed in all aa pairs that coevolved at a correlation >0.9. With respect to individual residues, aa residue at position 170 had the maximum number of five partners, of which, the most significant correlations (>0.9) were seen with aa residue 510(K) and 463(S).
- nsP3: nsP3 has 530 aa (aa) and the whole protein was taken for coevolution analysis. It was observed that 239 aa pairs involving 41 aa residues coevolved at a correlation >0.5, of which, 49 aa pairs showed a correlation of 0.9-1.0 (Fig. 2c). Mutations were seen in all aa pairs with a correlation of 0.9-1.0. With respect to individual residues, aa residue at position 361 had the maximum number of 18 partners, of which, the most significant correlations (>0.9) was seen with seven aa residue (Fig. 2c)
- nsP4: nsP4 has 661 aa (aa) and the whole protein was taken for coevolution analysis. It was observed that 46 aa pairs involving 21 aa residues coevolved at a correlation >0.5, of which, 19 aa pairs showed a correlation of 0.9-1.0 (Fig. 2d). It was observed that all these 19 aa pairs were found mutated with respect to the Delhi samples. With respect to individual residues, aa residue at position 15 had the maximum number of nine partners, of which, the most significant correlations (>0.9) was seen with five aa residue (Fig. 2d)
- Polyprotein: The entire polyprotein of the non-structural proteins has 2475 amino acid (aa) and was taken for coevolution analysis. It was observed that 705 aa pairs involving 138 aa residues coevolved at a correlation >0.5, of which, 232 aa pairs showed a correlation of 0.9-1.0 (Fig. 3). It is noteworthy that nsP3 has the highest number of coevolving residues i.e. 27 followed by 18, 16 and 13 coevolving residues in nsP4, nsP2 and nsP1 respectively. With respect to individual residues, aa residue at position 519 of nsP1 and at position 999 of nsP2 had the maximum number of 19 and 18 partners respectively coevolving at the most significant correlations (>0.9) (Fig. 3).

- nsP1: nsP1 has 535 aa (aa) and the whole protein was taken for coevolution analysis. It was observed that 30 aa pairs involving 23 aa residues coevolved at a correlation >0.5, of which, seven aa pairs showed a correlation of 0.9-1.0 (Fig. 2a). Mutations were seen in all aa pairs coevolving with a correlation of 0.9-1.0. With respect to individual residues, aa residue at position three had the maximum number of five partners, of which, the most significant correlation (>0.9) was seen with aa residue 472(R).

**Fig. 3:**
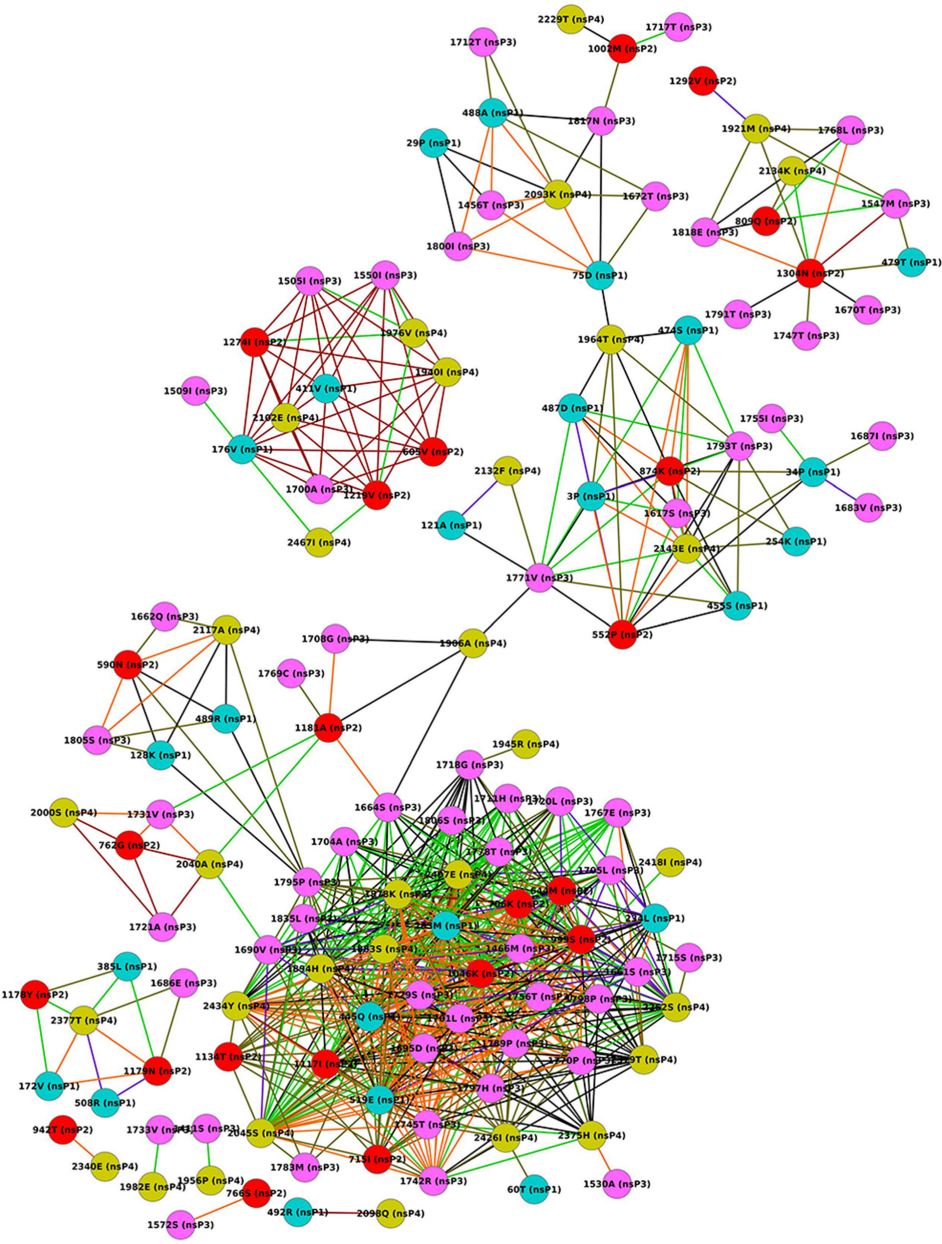
Coevolution analysis of non-structural polyprotein. Complete polyprotein was analyzed for inter-molecular coevolving aa residues using CAPS software and network was generated similarly using Cytoscape software. Edge color varies with correlation value: red (1.0), orange (~0.9), purple (~0.8), light green (~0.7), black (~0.6) and dark green (~0.5) and individual nsPs have been differently colored (nsP1: blue, nsP2: red, nsP3: pink, nsP4: green)

### ELM studies

The linear motifs in this study were predicted using The Eukaryotic Linear Motif resource for Functional Sites in Proteins web server. The ELM instances present in the database are classified under different classes. In total, there are 240 different classes of motif with 2700 experimentally validated classes, like cleavage sites (CLV), Degradation sites (DEG), Docking sites (DOC), Ligand binding sites (LIG), Post-translational modification sites (MOD) and Targeting sites (TRG). The ELMs of all non-structural proteins of CHIKV were predicted and further correlated with the intra-molecular coevolving sites of each non-structural protein. For the sake of stringency and functional relevance, coevolving sites with correlation cutoff value ≥ 0.9 were only used for deducing their functional significance. The results of the aa residues of coevolving pair and their respective ELMs are shown in Table 5a-d. After filtering, total of seven pairs of coevolving residues of nsP1, fifteen pairs of nsP2, 83 pairs of nsP3 and 27 coevolving pairs of nsP4 were used for analysis. It is interesting to note that nsP3 despite of being the smallest protein (530 aa) in comparison to other non-structural protein was showing high number of coevolving pairs; this is may be due to the presence of hyper variable region towards its C-terminal. Many common motifs were found between the aa residues of coevolving pair. With respect to nsP1, it was seen that 13 residues with correlation value > =0.9, showed ELMs with the humans (Table 5a). nsP2 has 13 residues (Table 5b), nsP3 showed 23 (Table 5c) and nsP4 has 14 residues (Table 5d) with correlation value above threshold and also has ELMs.

**Table 5:** The tables show the predicted ELMs for the coevolving residues along with the coevolving positions of amino acid residue of non-structural proteins of Chikungunya virus. The coevolving amino acid residues are written in the bracket “()”

a: ELMs for coevolving residues in nsP1 where,	
Position (residue)	ELMs^a^
3(S)	LIG_WD40_WDR5_VDV_1, LIG_LIR_Gen_1, DEG_Nend_UBRbox_2
75(D)	DRK,CLV_NRD_NRD_1
172(V)	DOC_MAPK_1
176(V)	DOC_MAPK_1
291(M)	MOD_ProDKin_1, MOD_PKA_1, MOD_PKA_1, DOC_WW_Pin1_4
383(L)	LIG_SH2_STAT5
409(V)	DOC_CYCLIN_1
453(G)	MOD_GSK3_1
472(R)	TRG_LysEnd_APsAcLL_1,DOC_CYCLIN_1, CLV_PCSK_SKI1_1
485(N)	MOD_GlcNHglycan
486(A)	MOD_GlcNHglycan
506(H)	DEG_APCC_DBOX_1
517(E)	LIG_TRAF2_1
b: ELMs for coevolving residues in nsP2	
Position (residue)	Motifs^b^
16(L)	MOD_GSK3_1,LIG_FHA_1
170(K)	LIG_SH3_1,LIG_SH3_3
273(L)	DOC_MAPK_1
338(M)	LIG_SUMO_SIM_par_1
463(S)	MOD_GSK3_1
510(K)	MOD_ProDKin_1,LIG_14-3-3_3,DOC_WW_Pin1_4
54(S)	LIG_Integrin_isoDGR_1
642(Y)	MOD_NEK2_1,MOD_GlcNHglycan
683(V)	LIG_LIR_LC3C_4,LIG_SUMO_SIM_anti_2
69(V)	TRG_LysEnd_APsAcLL_1,LIG_SH3_3,LIG_eIF4E_1
768(S)	MOD_NEK2_1
c: ELMs for coevolving residues in nsP3	
Position (residue)	Motifs^c^
122(T)	LIG_14-3-3_2,MOD_NEK2_1,MOD_PKA_2
132(M)	CLV_C14_Caspase3-7,MOD_GSK3_1
327(S)	LIG_TRAF2_1,MOD_CK1_1,MOD_CK2_1,MOD_GSK3_1,MOD_PKA_2
330(S)	MOD_GlcNHglycan,LIG_TRAF2_1,MOD_CK1_1,MOD_CK2_1,MOD_GSK3_1,MOD_PKA_2
332(Q)	MOD_GlcNHglycan,LIG_TRAF2_1,MOD_CK1_1,MOD_CK2_1,MOD_GSK3_1
349(V)	MOD_PLK
361(D)	CLV_C14_Caspase3-7
377(H)	MOD_GSK3_1
378(T)	MOD_GSK3_1
381(S)	MOD_GSK3_1
395(S)	LIG_SH3_3
397(V)	LIG_SH3_3
408(R)	CLV_PCSK_SKI1_1,MOD_N-GLC_1,DOC_CYCLIN_1,DOC_MAPK_1,MOD_PKB_1,TRG_ER_diArg_1
411(T)	CLV_PCSK_SKI1_1,MOD_N-GLC_1,DOC_CYCLIN_1,DOC_MAPK_1,MOD_CK2_1,MOD_PKB_1
434(L)	DEG_APCC_DBOX_1
436(P)	DEG_APCC_DBOX_1
437(A)	DEG_APCC_DBOX_1
452(Q)	DOC_PP2B_LxvP_1
455(P)	MOD_CK2_1
462(N)	LIG_SH3_3,MOD_CK1_1
463(H)	LIG_SH3_3
464(P)	LIG_EVH1_2,LIG_SH3_3,MOD_GSK3_1
466(I)	LIG_EVH1_2,MOD_GSK3_1
d: ELMs for coevolving residues in nsP4	
Position (residue)	Motifs^d^
113(V)	MOD_CK2_1,MOD_GSK3_1,MOD_PKA_2
137(S)	DOC_USP7_1,MOD_GSK3_1
15(K)	DOC_MAPK_1,MOD_GSK3_1,MOD_PKA_2
182(S)	LIG_14-3-3_3,MOD_CK1_1,MOD_GSK3_1
20(S)	DOC_MAPK_1,MOD_GSK3_1,MOD_PKA_2
512(H)	MOD_GSK3_1,MOD_NEK2_1
571(Y)	MOD_CK2_1
582(A)	LIG_SUMO_SIM_anti_2
603(V)	DOC_MAPK_1,MOD_NEK2_2
77(I)	LIG_Actin_WH2_2
79(R)	CLV_PCSK_FUR_1,DOC_MAPK_1,LIG_Actin_WH2_2
81(K)	CLV_PCSK_FUR_1,CLV_PCSK_PC1ET2_1,DOC_MAPK_1,LIG_Actin_WH2_2
90(A)	LIG_FHA_1,LIG_SH2_STAT5,LIG_SH3_3,MOD_ProDKin_1,DOC_WW_Pin1_4

^a^ *LIG_WD40_WDR5_VDV_1* WDR5 WD40 repeat (blade 5,6)-binding ligand, *LIG_LIR_Gen_1* Atg8 protein family ligands, *DEG_Nend_UBRbox_2* N-degron, *CLV_NRD_NRD_1* NRD cleavage site, *DOC_MAPK_1* MAPK docking motifs, *MOD_ProDKin_1* MAPK Phosphorylation Site, *MOD_PKA_1* PKA Phosphorylation site, *DOC_WW_Pin1_4* WW domain ligands, *LIG_SH2_STAT5* SH2 ligand, *DOC_CYCLIN_1* Cyclin recognition site, *MOD_GSK3_1* GSK3 phosphorylation site, *TRG_LysEnd_APsAcLL_1* Endosome-Lysosome-Basolateral sorting signals, *CLV_PCSK_SKI1_1* PCSK cleavage site, *MOD_GlcNHglycan* Glycosaminoglycan attachment site, *DEG_APCC_DBOX_1* APCC-binding Destruction motifs, *LIG_TRAF2_1* TRAF2 binding site

^b^ *MOD_GSK3_1* GSK3 phosphorylation site, *LIG_FHA_1* FHA phosphopeptide ligands, *LIG_SH3_1* SH3 ligand, *LIG_SH3_3* SH3 ligand, *DOC_MAPK_1* MAPK docking motifs, *LIG_SUMO_SIM_par_1* SUMO interaction site, *MOD_ProDKin_1* MAPK Phosphorylation Site, *LIG_14-3-3_3* 14-3-3 ligand, *DOC_WW_Pin1_4* WW domain ligands, *LIG_Integrin_isoDGR_1* Integrin binding sites, *MOD_NEK2_1* NEK2 phosphorylation site, *MOD_GlcNHglycan* Glycosaminoglycan attachment site, *LIG_LIR_LC3C_4* Atg8 protein family ligands, *LIG_SUMO_SIM_anti_2* SUMO interaction site, *TRG_LysEnd_APsAcLL_1* Endosome-Lysosome-Basolateral sorting signals, *LIG_eIF4E_1* eIF4E binding motif

^c^ *LIG_14-3-3_2* 14-3-3 ligand, *MOD_NEK2_1* NEK2 phosphorylation site, *MOD_PKA_2* PKA Phosphorylation site, *MOD_GSK3_1* GSK3 phosphorylation site, *LIG_TRAF2_1* TRAF2 binding site, *MOD_CK1_1* CK1 Phosphorylation site, *MOD_PLK* Plk phosphorylation site, *LIG_SH3_3* SH3 ligand, *MOD_N-GLC_1* N-glycosylation site, *DOC_CYCLIN_1* Cyclin recognition site, *MOD_PKB_1* PKB Phosphorylation site, *TRG_ER_diArg_1* di Arginine retention/retrieving signal, *DOC_PP2B_LxvP_1* Calcineurin (PP2B)-docking motif LxvP, *LIG_EVH1_2* EVH1 ligands

^d^ *MOD_CK2_1* CK2 Phosphorylation site, *MOD_GSK3_1* GSK3 phosphorylation site, *MOD_PKA_2* PKA Phosphorylation site, *DOC_USP7_1* USP7 binding motif, *LIG_14-3-3_3* 14-3-3 ligand, *MOD_CK1_1* CK1 Phosphorylation site, *MOD_NEK2_1* NEK2 phosphorylation site, *LIG_Actin_WH2_2* Actin-binding motifs, *CLV_PCSK_FUR_1* PCSK cleavage site, *LIG_FHA_1* FHA phosphopeptide ligands, *LIG_SH2_STAT5* SH2 ligand, *LIG_SH3_3* SH3 ligand, *MOD_ProDKin_1* MAPK Phosphorylation Site, *DOC_WW_Pin1_4* WW domain ligands

## Discussion

One approach to identify beneficial mutations is by studying coevolving aa that result in establishment of novel and divergent strains within a short period of time. Functional attributes of these coevolving aa are associated with their interactions with host proteins [27]. Much advances have been made in studying protein coevolution at the molecular level making use of both sequence information as well as protein structure information of the interacting proteins of both the host and the virus [28]. In case of CHIKV, as the complete structures of nsPs are presently not available, we made use of sequence information to deduce the coevolving aa within the individual nsPs and between the nsPs in the polyprotein. Clinical samples collected over three consecutive years after a major outbreak were studied for coevolving aa in their nonstructural proteins. Analyzing the coevolving aa on the basis of their correlation coefficient values, our results have revealed that aa pairs having more than 0.9 correlation value have lesser number of coevolving partners thereby emphasizing the stringency of the technique. Of special mention is the analysis of the coevolving aa in nsP3 sequences from across the globe. While analyzing this protein, we observed that there were a variety of deletions in aa positions 376-382. Eighteen sequences isolated in the year 2014, mainly from South America (KR264951.1-KR264951.1, KP851709.1-KP851710.1, KJ451624.1) and Micronesia (KJ689452.1-KJ689453.1, KJ451622.2-KJ451623.1) showed deletion of four aa in position 379-382, whereas one sample each from Germany (KM673291.1), New Caledonia (HE806461.1), China (KF318729.1), Indonesia (FJ807897.1) two from Malaysia (FN295483.3, FN295484.2) and three from Brazil (KP164570.1-KP164571.1, KP164567.1) collected between 2006-2013 showed deletions in position 376-382. GO term analysis of this region using Eukaryotic Linear Motifs (ELMs) revealed that this motif present in the hyper variable region of nsP3 might be playing a role in protein aa phosphorylation and could be important for virus stability. Wet lab experiments are required to clearly understand the importance of this deletion both to the virus and to the host.

**Fig. 4:**
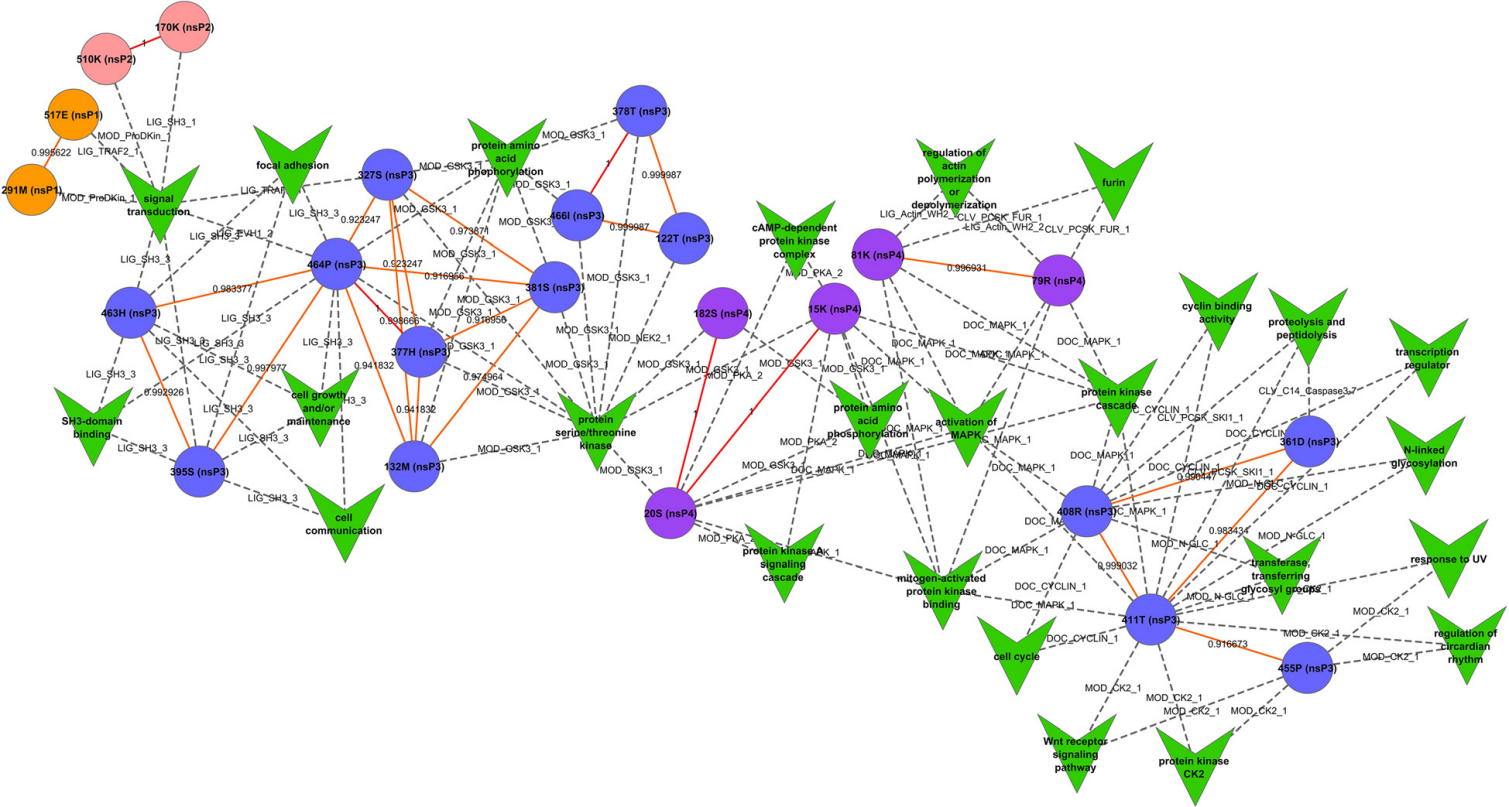
The figure represents the correlation between ELMs GO terms and coevolving pairs. The nodes are colored according to the respective protein and colored solid edges represent the correlation coefficient value. Values are also written along with the edge. Dash-lines represent the relation between coevolving pair and their respective ELM functions. The ELM names are also shown in the figure

Viruses are dependent on host factors and enzymes for their replication and other processes due to the lack of their own protein processing machinery. Utilizing ELMs for studying the functional relevance of the CHIKV nsPs coevolving aa revealed interesting insights to the interactions of these proteins with the host. A total of 23 aa residues (27 coevolving pairs) that showed correlation co-efficiency of 0.9-1.0 were analyzed for their functional significance. Network of coevolving aa pairs and their predicted functionality is represented in Fig. 4. Amino acid 464(P) of nsp3 was predicted to be coevolving with maximum number of partners, namely, six other residues of nsP3 protein. It was observed that most of these pairs (464(P)-377(H), 464(P)-132(M), 464(P)-381(S) and 464(P)-327(S) were associated with motifs that participated in protein aa phosphorylation and protein serine/threonine kinase activity and has been experimentally validated in other systems [29, 30]. Additionally, this residue when paired with 327(S) was predicted to play a role in signal transduction represented by motif LIG_EVH1_2 and LIG_TRAF2_1. Herpesviruses proteins have been shown to be recruited through these motifs in TGFβ signaling and NF kb activation [31, 32]. Pairing of this aa with residue 395S were seen to be involved in several functions, namely, cell communication, SH3-binding domain, focal adhesion and signal transduction. Other functions of the coevolving pairs of nsP3 include cell communication and cell growth and maintenance (395(S)- 463(H)), cell cycle, proteolysis and peptidolysis (361(D)-411(T), 361(D)-408(R)), Wnt receptor signaling pathway, regulation of circardian rhythm, response to UV, protein kinase CK2 activity (411(T)-455(P)). transferringglycosyl group and activation of MAPK pathway (411(T)-408(R)). Involvement of kinases in virus activation through these motifs have been studied in papillomaviruses [33, 34].

With respect to nsP4, three coevolving pairs (five aa residues) were found to participate in functions like furin process, regulation of actin polymerization and depolymerization (79(R)-81(K)), cAMP-dependent protein kinase complex (20(S)-15(K)) and protein aa phosphorylation (20(S)-15(K), 20(S)-182(S)) (Fig. 4). Both nsP1 and nsP2 has one pair each of coevolving aa residues sharing common functions. 291(M)-517(E) coevolving pair of nsP1 and 170(K)-510(K) coevolving residues of nsP2 were predicted to play a role in signal transduction process (Fig. 4).

## Conclusions

Our study has provided insight on the coevolving aa in the nonstructural proteins of CHIKV. Further, we have also attempted to predict the functional relevance of these aa with respect to the host using ELMs. It should be noted, however, correlation between coevolving pair of aa and ELMs in case of CHIKV require experimental validation.
